# Characterization of the complete chloroplast genome of *Ilex crenata* Thunb. (Aquifoliaceae)

**DOI:** 10.1080/23802359.2021.1961626

**Published:** 2021-08-09

**Authors:** Xinran Chong, Hong Chen, Chuanyong Wang, Ting Zhou, Yunlong Li, Yanwei Zhou, Ting Zhang, Xiaoqin Lu, Fan Zhang

**Affiliations:** Jiangsu Key Laboratory for the Research and Utilization of Plant Resources, Institute of Botany, Jiangsu Province and Chinese Academy of Sciences, Nanjing, China

**Keywords:** *Ilex crenata* Thunb., Aquifoliaceae, chloroplast genome, phylogenetic analysis

## Abstract

*Ilex crenata* Thunb. is a species of Aquifoliaceae with high ornamental and ecological values. In this study, the complete chloroplast (cp) genome of *I. crenata* was assembled and characterized through Illumina sequencing data. The entire cp genome of *I. crenata* was 157,988 bp in length with 37.64% overall GC content, containing a large single-copy (LSC) region of 87,414 bp and a small single-copy (SSC) region of 18,422 bp, which were separated by a pair of 26,076 bp inverted repeat (IR) regions. A total of 135 genes were annotated, including 88 protein-coding genes, 39 tRNA genes, and 8 rRNA genes. Phylogenetic analysis based on 78 conserved protein-coding genes demonstrated that *I. crenata* is closely related to *I. viridis* and *I. szechwanensis*.

*Ilex* (holly) is the largest woody dioecious genus in the angiosperms containing approximately 700 species within the monogeneric family of Aquifoliaceae (Su et al. 2020). The great diversity and adaptability of *Ilex* plants make them indispensable in gardens and landscapes. Among them, *Ilex crenata* Thunb. is native to eastern China, Japan and Korea, and has been widely utilized as an ornamental plant for its dense evergreen foliage and various forms (Dirr 2009). Many cultivars and hybrids have been bred based on this species (Yang et al. 2015). However, the genetic and genomic resources of the species are very limited. Here, we first assembled and characterized the complete chloroplast (cp) genome of *I. crenata* by Illumina sequencing and bioinformatics analysis, which will contribute to the further studies on the identification and phylogenetic analysis of *Ilex* species.

*I. crenata* were planted in Nanjing Botanical Garden, Mem. Sun Yat-sen (E118_83, N32_06), Nanjing, China. The voucher specimen (accession number NBGJIB-Ilex-0037) was stored at the Institute of Botany, Jiangsu Province and Chinese Academy of Science. Fresh leaves were snap-frozen in liquid nitrogen, and the total DNA was extracted from the frozen tissue using the GMS16011.2.1 Kit (Genmed Scientifics Inc., USA). A paired-end library with an insert-size of 350 bp was constructed and sequenced by the Illumina NovaSeq system (Illumina, San Diego, CA) at Novogene company (Tianjin, China). In total, 5607 Mb raw data (5438.4 Mb clean data) were generated. The filtered sequences were used for the *de novo* genome assembly by the program NOVOPlasty version 3.3 (Dierckxsens et al. 2017) and direct-viewing in Geneious R11 (Biomatters Ltd., Auckland, New Zealand). Annotation was performed using GeSeq with further manual correction (Tillich et al. 2017). The complete cp genome of *I. crenata* was deposited in GenBank database (accession number: MW528027).

In general, the whole cp genome of *I. crenata* was 157,988 bp with 37.64% GC content, including a large single-copy (LSC) region of 87,414 bp, a small single-copy (SSC) region of 18,422 bp and two inverted repeat (IR) regions of 26,076 bp. The cp genome contained 88 protein-coding genes, 39 tRNA genes, and 8 rRNA genes. Among them, 8 protein coding genes, 7 tRNA genes and 4 rRNA genes are duplicated in the IR regions. Moreover, fifteen genes contained one intron and three genes (*ycf3*, *clpP* and *rps12*) contained two introns.

Phylogenetic analysis was performed between *I. crenata* and other 14 *Ilex* species, with *Helwingia himalaica* used as an outgroup (Yao et al. 2016; Cascales et al. 2017; Park et al. 2019; Su et al. 2020). All the cp genome sequences other than *I. crenata* were downloaded from the NCBI database. The 78 common protein-coding genes were extracted and aligned using MAFFT v7.471 (Katoh et al. 2019) with default parameters, and then used for tree construction. Phylogenetic tree was constructed by the maximum likelihood (ML) method with the best fit model GTR + I + G using PhyML version 3.0 (Liu et al. 2019). The Best-fit model was tested according to the Akaike information criterion (AIC) by jModeltest version 2 (Guindon and Gascuel 2003; Darriba et al. 2012). The bootstrap values were calculated using 1000 replicates. The phylogenetic tree showed that *I. crenata* was clustered into the *Paltoria* section, and closely related to *I. viridis* and *I. szechwanensis* (Figure 1). The complete cp genome sequence of *I. crenata* will be useful for further analysis on genetic diversity, phylogeny, and molecular breeding.

**Figure 1.**
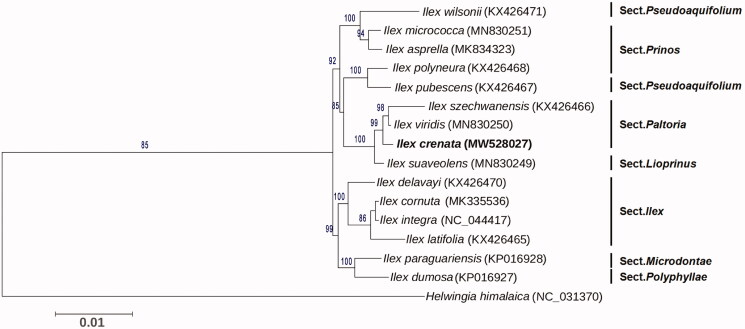
Maximum likelihood phylogenetic tree based on 78 protein-coding genes of *I. crenata* and other 15 species. Section names were displayed in the right side of phylogenetic tree. Numbers on the nodes indicated the bootstrap values. Genbank accession number of each species was shown in the brackets after names.

## Data Availability

The complete chloroplast genome sequence of *I. crenata* Thunb. is deposited in Genbank of NCBI (https://www.ncbi.nlm.nih.gov/) under the accession number MW528027. The associated BioProject, SRA, and Bio-Sample numbers are PRJNA690195, SRR13375982, and SAMN17245922, respectively.
